# Acetic Acid-Treated Common Beans Attenuate the Host Plant Preference of Spider Mites

**DOI:** 10.3390/plants15101460

**Published:** 2026-05-11

**Authors:** Kazuhiko Tamai, Kenji Matsui

**Affiliations:** 1 Research & Development Headquarters, Earth Corporation, Ako-shi 678-0192, Japan; 2 Graduate School of Sciences and Technology for Innovation (Agriculture), Yamaguchi University, Yamaguchi 753-8515, Japan

**Keywords:** vinegar, pesticides, acetic acid, volatiles, *Phaseolus vulgaris*, *Tetranychus urticae*

## Abstract

Chemical pesticides are indispensable for protecting crops from pests and supporting a stable food supply; however, their environmental impacts have raised growing concerns. This has driven efforts to develop alternative, lower-impact pesticides and technologies for sustainable agriculture. Vinegar, a natural and eco-friendly option, has been used to protect plants from pests and diseases. Here, we investigated the effects of acetic acid, the main component of vinegar, on host selection by spider mites using a common bean (*Phaseolus vulgaris*)-two-spotted spider mite (*Tetranychus urticae*) system. The results indicated that spider mites avoided acetic acid-treated plants, with plant volatiles influencing their preference. Volatile organic compound (VOC) analysis revealed that acetic acid treatment altered VOC composition. Although changes in the levels of compounds such as (*E*)-2-hexenal and 1-octen-3-one contributed to this shift, individual testing showed that unrealistically high concentrations were required to repel spider mites. This suggests that the effect of acetic acid treatment is not primarily due to the induction of specific VOCs, but rather to an overall alteration of VOC composition. These findings highlight the potential of vinegar as an eco-friendly pest management strategy, although further studies are needed to evaluate its long-term efficacy and impact on crop protection under field conditions.

## 1. Introduction

Current agricultural practices heavily rely on synthetic pesticides for pest control [1], an indispensable task for ensuring a stable food supply. However, a global push towards sustainable agriculture has initiated efforts toward reducing this dependency as the overuse of synthetic pesticides sometimes has deleterious effects on the environment and biodiversity. Integrated pest management (IPM) has been proposed as an effective strategy to meet the increasing demand for food associated with rapid population growth while mitigating the drawbacks of synthetic pesticides. IPM requires optimised crop management based on a thorough understanding of crop–pest interactions and the appropriate integration of biological and chemical control methods [2]. In addition, the development of pesticides with higher degradability, greater target specificity, and improved environmental compatibility is being actively pursued for use in chemical control [3]. In this context, increasing attention has been paid to biostimulants, which enhance the inherent resistance of plants rather than directly killing pests [4]. Among them, acetic acid has recently emerged as a promising candidate. Historically, acetic acid has been used as a herbicide at relatively high concentrations (10–20%) [5]. However, recent reports have shown that acetic acid treatment of plants at low concentrations improves plant tolerance to abiotic stresses [6]. In *Arabidopsis thaliana*, irrigation of soil in pots with 0–50 mM (0–0.3%) acetic acid significantly increased desiccation tolerance [7]. Further, spraying 20 mM (0.12%) acetic acid on the leaves of mung bean (*Vigna radiata*) seedlings enhanced growth and mitigated seawater-induced salt toxicity [8]. Enhanced salt stress tolerance has also been reported in canola [9]. In contrast, although it has been empirically suggested that acetic acid can enhance plant resistance to biotic stress [10], relatively few studies have addressed this aspect. To date, only a limited number of reports are available. Acetic acid has been shown to prime resistance in tomato (*Solanum lycopersicum*) shoots against larvae of the tobacco cutworm (*Spodoptera litura*) [11]. In addition, acetic acid treatment has been reported to induce resistance to root-knot nematodes in tomato roots and to enhance resistance to Fusarium wilt disease in tomato plants [12,13].

Two-spotted spider mite (*Tetranychus urticae* Koch) is a polyphagous, cell-content-sucking herbivore that is a long-standing and crucial target for pest control as it significantly reduces crop yield and quality [14]. Spider mites are highly prone to pesticide resistance development due to characteristics like male-producing parthenogenesis, high reproductive capacity, short generation time, inbreeding, and high xenobiotic metabolism capabilities [15]. Therefore, new pest control technologies are in high demand. If acetic acid treatment can enhance plant resistance to spider mites, it could provide a novel and sustainable approach for controlling mite infestations.

In this study, we applied acetic acid treatment to common bean (*Phaseolus vulgaris*) seedlings and observed the behaviour of the two-spotted spider mite (*T. urticae*). Because mite settlement was suppressed in acetic acid-treated *P. vulgaris* seedlings, we analysed the volatile organic compound (VOC) profile to clarify the effects of acetic acid treatment. The results showed that acetic acid treatment altered the composition of emitted volatiles, which may have contributed to the suppression of mite settlement.

## 2. Results

**Figure 1:**
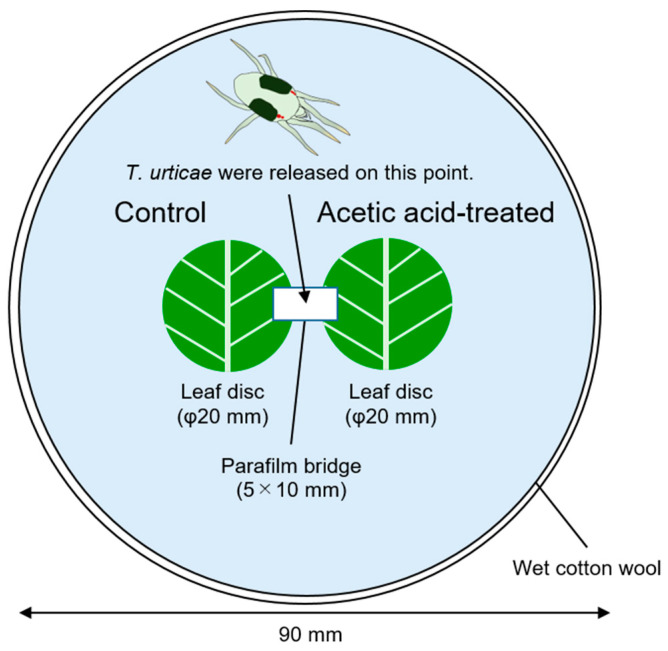
Setup for testing the preference of *Tetranychus urticae* for acetic acid-treated leaf discs. Leaf discs prepared from acetic acid (10, 20, or 40 mM)-treated plants and leaf discs prepared from control plants (0 mM acetic acid) were placed on Petri dishes paved with wet cotton. The leaf discs were bridged with Parafilm (5 × 10 mm), and one *T. urticae* individual was released at the centre of the Parafilm bridge. After 24 h, the leaf disc on which the individual was found was recorded as the preferred leaf disc.

**Figure 2:**
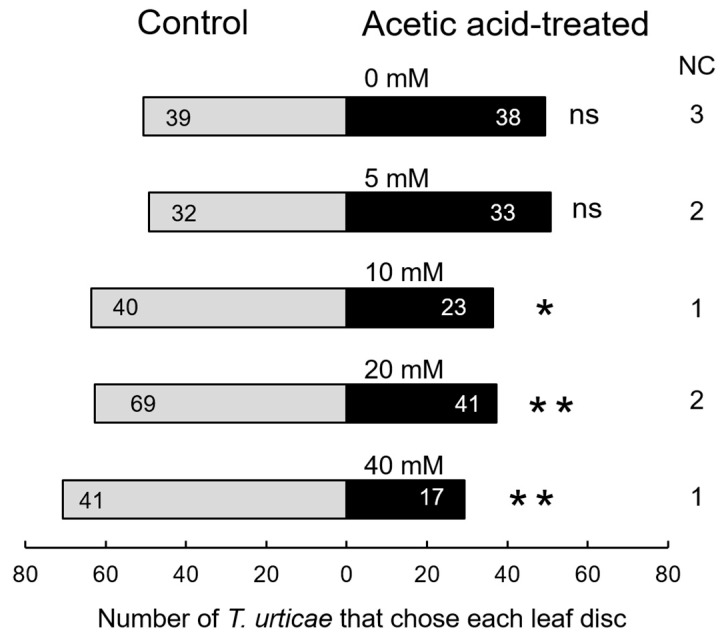
Response of *Tetranychus urticae* to leaf discs from plants treated with different acetic acid concentrations. Numbers in the bar segments indicate the number of *T. urticae* that chose each leaf disc. NC, number of *T. urticae* found outside the leaf discs; *, significant difference between treatments (chi-square test: * *p* < 0.05, ** *p* < 0.01, ns = *p* > 0.05).

First, spider mite preference was investigated in a behavioural observation experimental system using acetic acid-treated common bean leaf discs. Ten-day-old *P. vulgaris* seedlings were sprayed with acetic acid (dissolved in 0.05% Silwet, added as a surfactant) or a control solution (0.05% Silwet without acetic acid) twice at a 48 h interval. Three days after the second application, leaf discs 20 mm in diameter were cut and placed in a 90 mm Petri dish arena together with a control disc, and a bridge made of Parafilm was set between them (Figure 1). Spider mites were placed at the centre of the bridge, and after 24 h, the number of mites that had settled on each leaf disc was counted. Under these experimental conditions, spider mites did not discriminate between leaf discs prepared from 5 mM acetic acid-treated plants and 0 mM control discs. However, they did significantly discriminate between 0 mM control discs and leaf discs prepared from 10 mM acetic acid-treated leaves and were repelled by the 10 mM acetic acid-treated leaf discs (Figure 2). Significant repulsion was also observed for 20 and 40 mM acetic acid-treated leaf discs, confirming that spider mites were repelled by bean leaf discs treated with more than 10 mM acetic acid.

**Figure 3:**
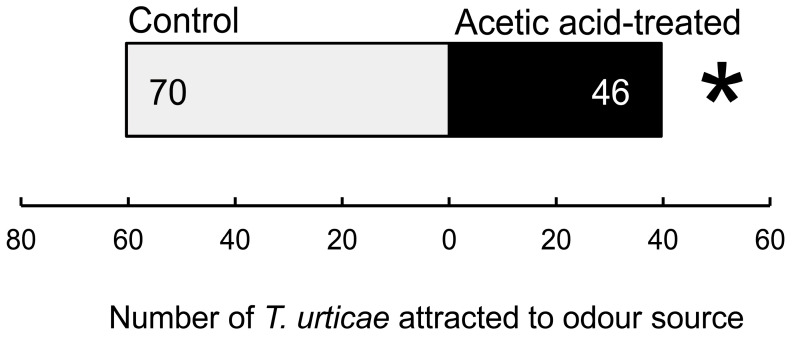
Response of *Tetranychus urticae* toward volatiles from 40 mM acetic acid-treated and control plants. Numbers in bar segments indicate the number of *T. urticae* that chose each odour source. Twenty-one individuals did not reach either end of the Y-tube olfactometer within 15 min. *, significant difference between the treatments (chi-square test: * *p* = 0.03).

To determine whether volatile compounds emitted from acetic acid-treated common bean leaves are responsible for this repulsion, a Y-tube olfactometer assay was performed. In this experiment, a 40 mM acetic acid solution was evenly sprayed onto the leaves of 10-day-old common bean seedlings, which were then placed in one of the two inlets of the Y-tube; the other side contained bean plants similarly treated with a control solution containing only 0.05% Silwet. To clearly demonstrate the effect of acetic acid treatment, 40 mM—at which the most pronounced effect was observed in the leaf disc assay—was used. The spider mites were placed at the entrance of the Y-tube and allowed to walk upwind to select an odour source at the junction point. The behaviour of 137 spider mites was observed, and 21 showed no choice. Spider mites selected the odour from control common beans significantly more frequently than that from the acetic acid-treated beans (Figure 3).

**Figure 4:**
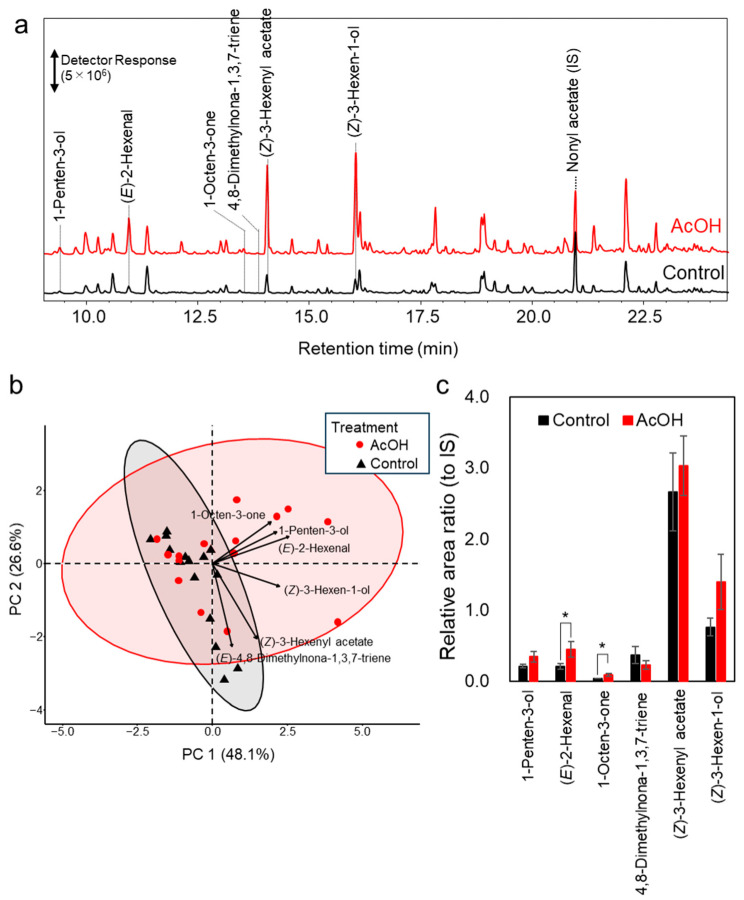
Volatile organic compounds (VOCs) emitted from acetic acid-treated *Phaseolus vulgaris* seedlings. Acetic acid (AcOH)-treated *P. vulgaris* seedlings were enclosed in a glass container, and air was passed through the chamber for 3 h to collect VOCs on Tenax, followed by analysis by thermal desorption–gas chromatography–mass spectrometry (TD–GC–MS). (**a**) GC–MS chromatograms of VOCs emitted from acetic acid-treated seedlings (red) and control seedlings treated with a solution lacking acetic acid (black). Among the major peaks, compounds considered to be emitted from common bean plants are annotated. Of these, (*E*)-4,8-dimethylnona-1,3,7-triene was tentatively identified based on mass spectral data. IS, internal standard. (**b**) Principal component analysis (PCA) biplot of volatile organic compounds. The score plot of samples (black: control; red: AcOH-treated) is combined with loading vectors representing individual compounds. PC1 and PC2 explained 48.1% and 26.6% of the total variance, respectively. The direction and length of each vector indicate the contribution of the corresponding compound to the principal components. (**c**) Differences in the emission levels of volatile compounds between control (black) and acetic acid-treated (red) plants. Compounds with peak area ratios to the internal standard (IS) below 0.05 were excluded. Values represent means ± standard error (SE) (*n* = 15). Asterisks indicate significant differences between groups as determined by Student’s *t*-test (*p* < 0.05).

These results suggest that VOCs may contribute to the repellence of spider mites from acetic acid-treated bean leaves. VOCs emitted from acetic acid-treated leaves were collected using a Tenax adsorbent and analysed by thermal desorption–gas chromatography–mass spectrometry. Under the present analytical conditions, several fatty acid-derived oxylipin volatiles, including 1-penten-3-ol, (*E*)-2-hexenal, 1-octen-3-one, (*Z*)-3-hexenyl acetate, and (*Z*)-3-hexen-1-ol, as well as the homoterpene, (*E*)-4,8-dimethylnona-1,3,7-triene, were detected (Figure 4a). Volatile collection and analysis were repeated five times on different days using 40 mM acetic acid-treated and control-treated common bean seedlings, with three biological replicates per treatment (control and acetic acid) conducted on each day. Because the differences in VOC profiles were not readily distinguishable by visual inspection, principal component analysis (PCA), an unsupervised multivariate analysis method, was performed. The PCA revealed that, although partial overlap was observed, the VOC profiles emitted from acetic acid-treated *P. vulgaris* exhibited distinct distribution trends compared with those of control plants, shifting toward the first quadrant (Figure 4b). The compounds contributing to this separation were 1-octen-3-one, (*E*)-2-hexenal, and 1-penten-3-ol. Among these, 1-octen-3-one and (*E*)-2-hexenal showed significant increases in relative peak area ratios following acetic acid treatment (Figure 4c, Table 1).

**Figure 5:**
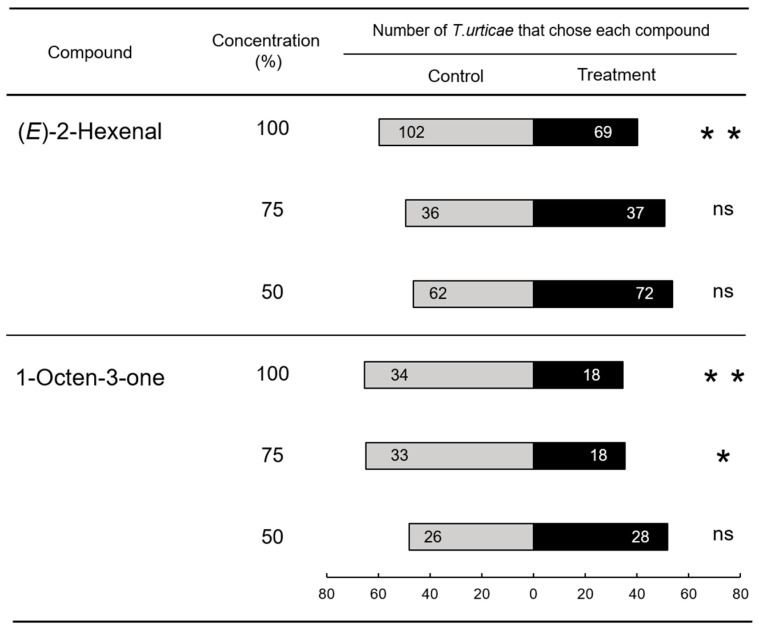
Response of *Tetranychus urticae* to volatile compounds from acetic acid-treated plants. Numbers in the bar segments indicate the number of *T. urticae* that chose each compound. *, significant difference between treatments (chi-square test: * *p* < 0.05, ** *p* < 0.01, ns = *p* > 0.05).

Next, we evaluated the preference of spider mites for these two oxylipin volatiles, 1-octen-3-one and (*E*)-2-hexenal, using a Y-tube olfactometer assay. The results showed that the two components showed no significant repellent effect at lower concentrations, and it was found that observing a pronounced repellency effect required extremely high concentrations of each compound in the Y-tube olfactometer, such as the undiluted compound or a 1:2 dilution (Figure 5).

## 3. Discussion

Acetic acid, a major component of vinegar, is produced by acetic acid bacteria and has long been utilised by humans as a fermentation product. Because acetic acid bacteria commonly colonise plant leaf and fruit surfaces [16], plants have likely been exposed to acetic acid throughout their evolutionary history. It is therefore reasonable to assume that many plant species have acquired the capacity to respond to exogenous acetic acid [6]. Indeed, low concentrations of acetic acid enhance drought tolerance in *Arabidopsis* and several crop species, including maize, rice, wheat, and rapeseed, suggesting a broadly conserved response mechanism [7]. In recent years, an increasing number of studies have demonstrated that acetic acid treatment enhances plant tolerance to abiotic stress. In contrast, although it has been empirically recognised that acetic acid treatment can suppress herbivory in crops [10], there are very few studies that clearly demonstrate whether acetic acid treatment alters plant resistance to biotic stresses such as herbivores and pathogens.

Given its growth-regulatory effects, environmental compatibility, and relatively low ecological impact, acetic acid has attracted attention as a biostimulant capable of inducing resistance to biotic stresses as well as abiotic stresses. Notably, acetic acid at concentrations below 8% is exempt from pesticide registration by the U.S. Environmental Protection Agency [6], highlighting its regulatory acceptance and potential applicability in organic agriculture. However, evidence supporting its role in enhancing resistance to biotic stress remains limited.

Using a common bean (*P. vulgaris*)-two-spotted spider mite (*T. urticae*) system, we demonstrated in this study that acetic acid treatment reduced spider mite settlement on bean leaves. It has been documented that treatment of plants with agrochemicals such as jasmonic acid and benzo(1,2,3)thiadiazole-7-carbothioic acid *S*-methyl ester (BTH) enhances resistance to spider mites [17,18]. However, to our knowledge, the suppression of spider mite behaviour by treatment with eco-friendly compounds such as acetic acid has not been previously reported. Further studies using a wider range of plant species and herbivores will be necessary to evaluate the general applicability and effectiveness of acetic acid. Plant-derived essential oils are also known to exhibit toxicity against spider mites [19]. Therefore, combining acetic acid-induced enhancement of plant defences with the application of essential oils as acaricides may provide a promising strategy for developing IPM approaches to reduce spider mite damage.

Y-tube olfactometry assays and VOC analyses suggested that this behavioural effect was accompanied by changes in the composition of volatiles. Thus, acetic acid-induced modulation of VOC profiles may contribute, at least in part, to reduced spider mite establishment. Spider mite host selection is influenced by multiple plant traits, including nutritional and defensive metabolites, leaf surface characteristics, and interactions with natural enemies [20]. In addition, spider mites use plant-emitted VOCs as olfactory cues, although their responses to VOC blends are complex. For example, spider mites avoid VOCs emitted from conspecific-infested bean plants and can discriminate between VOCs from spider mite-infested cucumber plants and thrip-infested plants, preferentially avoiding the latter [21]. Spider mites have also been reported to discriminate among lima bean leaves that differ in the extent of herbivore damage. However, in such cases, no significant differences have been detected in the emission levels of individual VOCs from the leaves [22]. These findings in previous studies suggest that spider mites do not rely on the quantity of specific compounds, but rather perceive subtle changes in the composition of the VOC bouquet. Consistent with this idea, adult male spider mites are known to be more strongly attracted to females guarded by another male than to solitary females [23]. In this case, no significant differences have been observed in the amounts of individual VOCs emitted from guarded versus solitary females. However, differences can be detected using Orthogonal Projection to Latent Structures Discriminant Analysis (OPLS-DA), a supervised multivariate analysis method [23]. This further supports the notion that spider mites respond to slight compositional variations in complex VOC blends rather than to changes in single compounds. Such integrative recognition of host-derived VOC blends during host location has been reported not only in spider mites but also in a wide range of herbivores [24].

In the present study, acetic acid treatment induced modest changes in VOC composition. The amounts of (*E*)-2-hexenal and 1-octen-3-one consistently increased; however, these increases were not the sole reason for spider mite avoidance of acetic acid-treated leaves. Indeed, concentrations several orders of magnitude higher than those likely emitted from acetic acid-treated leaves were required to elicit repellency when these compounds were tested individually. The difference in VOC composition between acetic acid-treated and control *P. vulgaris* plants was visualised by PCA. Although (*E*)-2-hexenal and 1-octen-3-one contributed to this separation, spider mites likely respond not to changes in individual compounds per se, but rather to subtle shifts in the overall VOC blend. How spider mites discriminate such subtle differences in VOC composition remains unclear. Therefore, to determine whether acetic acid-induced changes in VOC composition effectively reduce host preference under field conditions, experimental systems that evaluate behavioural responses to realistic VOC blends with subtle quantitative differences will be required. Furthermore, elucidating which defence pathways in *P. vulgaris* are affected by acetic acid treatment, leading to changes in VOC composition, remains an important subject for future research.

## 4. Materials and Methods

### 4.1. Mites and Plants

*T. urticae* were obtained in 2019 from a culture maintained at Chiba University Matsudo Campus (Matsudo, Japan) and maintained on common bean (*P. vulgaris* cv. Nagauzura) plants at 25 ± 1 °C, 60 ± 10% relative humidity (RH), and 16 L:8 D conditions. Non-starved adult females were randomly selected from the cultures for experiments conducted under the same conditions. *P. vulgaris* (cv. Nagauzura) plants used in the experiments were grown using commercial soil (Ikubyo-Baido, Takii, Kyoto, Japan) in a plastic pot (7.5 cm diameter × 6.5 cm height) for 10 days at 25 °C and 16 L (starting at 8 am, 266 µmol m^−2^ s^−1^)/8 D photo regime.

### 4.2. Acetic Acid Treatment

Acetic acid solution (5, 10, 20, and 40 mM in 0.05% Silwet L-77; Bio Medical Science Inc., Tokyo, Japan) was evenly sprayed on the above-ground portions of 10-day-old common bean seedlings until runoff (approximately 5 mL per plant) at 11 am. Excess acetic acid solution accumulated in leaf depressions was carefully removed using a paper towel. The treated plants were then allowed to grow under the same conditions as described previously. Acetic acid spraying was repeated after 48 h. Control plants were only sprayed with 0.05% Silwet L-77 solution.

### 4.3. Preference of T. urticae for Acetic Acid-Treated Leaf Discs

To test the preference of *T. urticae* for acetic acid-treated leaf discs, leaf discs were prepared exactly 72 h after the second acetic acid application to evaluate the induced plant response rather than the direct odour of the treatment. Leaf discs prepared from acetic acid-treated plants (5, 10, 20, and 40 mM acetic acid) and from control plants (i.e., 0% acetic acid) were placed 5 mm apart at the centre of a 9 cm Petri dish paved with wet non-fat cotton (Figure 1). The leaf discs were placed with their abaxial sides facing upward. Leaf discs were connected with a Parafilm^®^ bridge (Bemis Company, Neenah, WI, USA) (5 × 5 mm), and one spider mite was placed at the centre of the Parafilm bridge. The spider mite was able to freely choose between the two leaf discs via the bridge. Twenty-four h after spider mite inoculation, the leaf disc on which the spider mite was finally positioned was recorded as its choice. This experimental design was selected based on the biological characteristics of *T. urticae* to ensure the observation of stable host choice rather than transient exploratory behaviour, a methodology whose validity and efficacy have been well demonstrated in previous studies [25]. Mites were tested individually to eliminate the influence of social interactions, such as aggregation pheromones. Furthermore, to strictly prevent any potential cross-contamination or residual behavioural cues, the entire experimental arena—including the Petri dish, wet cotton, and Parafilm bridge—along with the leaf discs, was replaced with entirely fresh materials for each individual mite tested. To maintain optimal physiological conditions and experimental consistency, bioassays were limited to approximately 12 replicates per day and were systematically repeated over 38 independent days. A 24 h period was allowed for the mites to fully explore the options and settle, thereby avoiding accidental arrestment influenced by fine-scale leaf surface structures. Individuals that failed to reach either leaf disc within 24 h were excluded from the analysis to ensure that our statistical results reflected only active and deliberate host selection. The numbers of spider mites choosing each of leaf disc were compared using the chi-square test.

All statistical analyses were performed using R version 4.4.1 [26]. This applied to all subsequent statistical analyses presented in the study. Mites whose final position were not on the leaf discs were excluded from the statistical analysis. The 5, 10, 20, and 40 mM acetic acid treatments and the control treatment were repeated 67, 64, 112, 59, and 80 times, respectively.

All bioassays were conducted in a climate-controlled room at 25 ± 1 °C and 60 ± 10% RH, under uniform overhead LED lighting.

### 4.4. Preference of T. urticae for the Odour of Acetic Acid-Treated Plants

To test spider mite preference for the odour of acetic acid-treated plants or control plants, a horizontal glass Y-tube olfactometer [27] was used. Each odour source was set up with two plants exactly 72 h after the final acetic acid treatment. Purified air was pushed through the odour sources into both arms of the Y-tube at a constant rate of 4.0 L min^−1^ per arm. An iron Y-shaped wire was set at the centre of the Y-tube. One spider mite was individually positioned at the start position, the lower end of the iron Y-shaped wire. When the mite reached the end of one of the arms of the olfactometer, this was recorded as a choice. After recording, the mite was removed, the iron wire was cleaned, and a new test individual was introduced. To eliminate positional effects, the placement of acetic acid-treated and control plants was randomised. Spider mites that failed to choose an arm within the 15 min observation period were omitted from the statistical analysis. After 5–7 choice tests, the two odour sources were interchanged to adjust for potential asymmetries in the experimental arena. In total, 10–14 mites were used per replicate experiment. To confirm the absence of directional bias in our olfactometer setup and to ensure that the solvent itself had no effect on mite behaviour, preliminary negative control assays (clean air vs. clean air) were conducted. No significant differences in mite choice were observed (Supplementary Figure S1). The numbers of spider mites choosing each odour source were compared using the chi-square test. Mites that did not make a choice within 15 min were excluded from the statistical analysis. The experiment was replicated 132 times.

### 4.5. Volatile Analysis

VOCs produced by the plants were collected and characterised using dynamic headspace analysis. Ten-day-old *P. vulgaris* seedlings were sprayed twice with 40 mM acetic acid (containing 0.05% Silwet) under the conditions described above, with a 48 h interval between applications. Control plants were treated with 0.05% Silwet solution without acetic acid. VOC collection was initiated 72 h after the second application. The soil and pots containing the prepared plants were carefully wrapped in aluminium foil, leaving the above-ground parts open to the air, and one plant was set in each 2 L separable glass flask. The inlet flow was controlled using a flowmeter (Model RK20TM, Kofloc, Kyoto, Japan) at 0.3 L min^−1^ and passed through an ORBO 32 activated charcoal filter (SUPELCO, Bellefonte, PA, USA). The air was then ejected through a Tenax TA thermal desorption tube (60/80 mesh 150 mg, GL Science, Tokyo, Japan), using a diaphragm vacuum pump (DAP-6D, Ulvac Kiko, Miyazaki, Japan) at 0.3 L min^−1^ set using a flowmeter. Before volatile collection, the Tenax tube was conditioned using a Sample Tube Conditioner (SC4200, GL Science) with a temperature programme starting at 40 °C (15 min) to 100 °C (15 min) at 10 °C min^−1^, then heated to 280 °C (120 min) at 10 °C min^−1^. Nitrogen gas was used at 100 mL min^−1^ for each tube. The sampling time was 180 min starting at 11 am.

VOC desorption was carried out using a TD-30R thermal desorber (Shimadzu, Kyoto, Japan). Prior to analysis, 1 µL of an acetone solution containing *n*-nonanyl acetate (1 µg µL^−1^; Tokyo Chemical Industry, Tokyo, Japan) was added as an internal standard using a microsyringe. The internal standard was then drawn through the adsorbent using an automated gas sampling pump (GSP-300FT-2; Gastec Co., Kanagawa, Japan) at 100 mL min^−1^ for 5 min. Volatile compounds were desorbed under a 70 mL min^−1^ flow of He gas at 250 °C for 10 min and then cryofocused on a Tenax TA trap at −25 °C. The trap was heated to 250 °C for 2 min. The desorbed volatile compounds were transferred to a gas chromatography–mass spectrometer (QP-5050; Shimadzu, Kyoto, Japan) with a 0.25 mm internal diameter × 30 m DB-WAX column (film thickness, 0.25 μm; Agilent, Santa Clara, CA, USA) through a fused-silica line heated to 220 °C. The column temperature was programmed as follows: 40 °C for 1 min, increasing by 5 °C min^−1^ to 220 °C, and then 220 °C for 5 min. The carrier gas (He) was delivered at a constant pressure of 61.8 kPa. The mass detector was operated in the electron-impact mode with an ionisation energy of 70 eV. The volatile compounds mentioned in this study except for (*E*)-4,8-dimethylnona-1,3,7-triene were confirmed by comparing their retention times and mass spectra with those of standard compounds. (*E*)-4,8-dimethylnona-1,3,7-triene was tentatively identified by comparing its mass spectral profile with those in the NIST23 library. (*E*)-2-Hexenal and 1-octen-3-one were quantified based on calibration curves constructed using *n*-nonanyl acetate as an internal standard. The other compounds were expressed as the ratio of the peak area of each compound to that of the IS obtained by GC–MS. VOC analyses were performed with three biological replicates per day for both the acetic acid-treated and control groups, and this experiment was repeated on five different days. A total of 15 independent measurements were obtained and analysed for each treatment group. PCA of VOC composition was performed using R version 4.4.1 [26]. Compounds (1-penten-3-ol, (*E*)-2-hexenal, 1-octen-3-one, (*E*)-4,8-dimethylnona-1,3,7-triene, (*Z*)-3-hexenyl acetate, and (*Z*)-3-hexen-1-ol) used for PCA were selected based on chromatographic data as they were derived from *P. vulgaris* leaves and exhibited a peak area ratio to IS of ≥0.05.

### 4.6. Preference of T. urticae for (E)-2-Hexenal and 1-Octen-3-One

The preference of *T. urticae* for varying concentrations of (*E*)-2-hexenal and 1-octen-3-one was evaluated using the same experimental methodology described in Section 4.4.

For the odour sources, (*E*)-2-hexenal and 1-octen-3-one were individually diluted to 100, 75, and 50% concentrations in triethyl citrate. A 0.2 mL aliquot of each concentration was dispensed into 2 mL amber glass vials (11.6 × 32 mm; Sigma, St. Louis, MO, USA). The vials were then sealed with open screw caps containing a PTFE/rubber septum pierced with a 2 µL micropipette (Drummond, Millan SA, Geneva, Switzerland) to control release, following a method similar to that described by Hu et al. [28]. Preliminary negative control assays confirmed that *T. urticae* did not differentiate between clean air and the solvent, nor between varying concentrations of triethyl citrate (Supplementary Figure S1).

The numbers of spider mites choosing each odour source were compared using the chi-square test. The (*E*)-2-hexenal 100, 75, and 50% treatments were repeated 178, 75, and 137 times, respectively, while the 1-octen-3-one 100, 75, and 50% treatments were repeated 56, 54, and 55 times, respectively.

## 5. Conclusions

This study showed that acetic acid treatment of *P. vulgaris* reduces host acceptance by the two-spotted spider mite (*T. urticae*). Behavioural assays and olfactometry indicate that this effect is associated with changes in plant-emitted VOCs. Although acetic acid alters VOC composition, individual compounds such as (*E*)-2-hexenal and 1-octen-3-one do not fully explain the observed repellence at biologically relevant levels, suggesting that spider mites respond to overall VOC blend changes. These findings support the potential use of acetic acid as an environmentally compatible tool in integrated pest management, although further validation under field conditions is required.

## Figures and Tables

**Table 1:** Changes in the amounts of (*E*)-2-hexenal and 1-octen-3-one emitted from acetic acid-treated or control *Phaseolus vulgaris* seedlings.

	Control	Acetic Acid
	(ng/3 h/plant)	
(*E*)-2-Hexenal	2.77 ± 0.47	6.39 ± 1.55 *
1-Octen-3-one	0.27 ± 0.03	0.73 ± 0.13 *

Average ± SE (*n* = 15) is shown. *, Significant difference (*p* < 0.05); Students’ *t*-test.

## Data Availability

Raw data were generated at Earth Corporation and Yamaguchi University. Derived data supporting the findings of this study are available from the corresponding author on request. The data are not publicly available due to corporate confidentiality.

## Supplementary Materials

The following supporting information can be downloaded at: https://www.mdpi.com/article/10.3390/plants15101460/s1, Figure S1: Responses of *Tetranychus urticae* in negative control Y-tube olfactometer assays.

## Abbreviations

The following abbreviations are used in this manuscript:

JA	Jasmonic acid
JA-Ile	Jasmonoyl-isoleucine
SA	Salicylic acid
VOCs	Volatile organic compounds
