# Supplementary material for: Acetic Acid-Treated Common Beans Attenuate the Host Plant Preference of Spider Mites
Source: Plants (Basel). 2026 May 11;15(10):1460. doi: 10.3390/plants15101460 (PMC13210600; doi:10.3390/plants15101460)
Supplement: Supplementary file 1 [file plants-15-01460-s001.zip › plants-4255636-supplementary.pdf]

Supplementary Materials

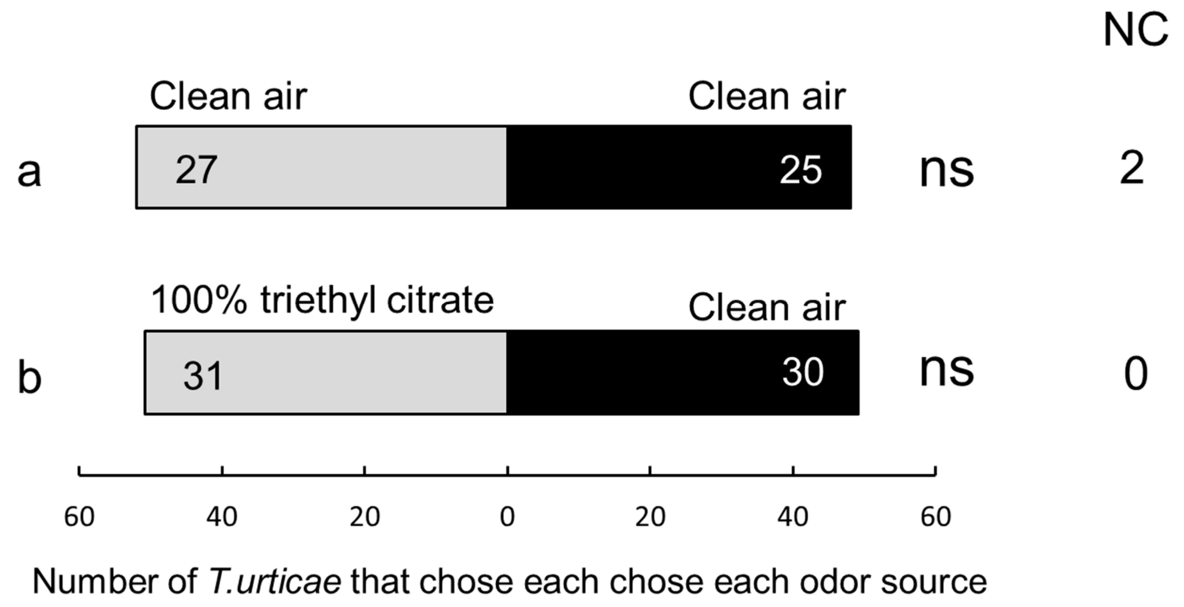

Figure S1. Responses of *Tetranychus urticae* in negative control Y-tube olfactometer assays.
To confirm the absence of directional bias in the experimental setup and to ensure that the solvent used in the chemical assays did not affect mite behavior, preliminary two-choice assays were conducted. The bars show the number of T. urticae females that chose each odor source. (a) Clean air versus clean air. (b) 100% triethyl citrate versus clean air. Numbers inside the bar segments indicate the total number of mites that made a choice. No significant differences were observed in any of the negative control assays (chi-square test: ns = $P > 0.05$)., indicating that the olfactometer setup was unbiased and the solvent itself had no effect on mite preference.
